# Definitive intensity modulated proton re-irradiation for lung cancer in the immunotherapy era

**DOI:** 10.3389/fonc.2022.1074675

**Published:** 2023-01-17

**Authors:** James R. Janopaul-Naylor, Yichun Cao, Neal S. McCall, Jeffrey M. Switchenko, Sibo Tian, Haijian Chen, William A. Stokes, Aparna H. Kesarwala, Mark W. McDonald, Joseph W. Shelton, Jeffrey D. Bradley, Kristin A. Higgins

**Affiliations:** ^1^ Winship Cancer Institute, Department of Radiation Oncology, Emory University School of Medicine, Atlanta, GA, United States; ^2^ Biostatistics Shared Resource, Winship Cancer Institute, Emory University, Atlanta, GA, United States; ^3^ Rollins School of Public Health, Department of Biostatistics and Bioinformatics, Emory University, Atlanta, GA, United States

**Keywords:** lung cancer, re-irradiation, proton therapy, immunotherapy, bronchial necrosis, radiation dermatitis

## Abstract

**Introduction:**

As immunotherapy has improved distant metastasis-free survival (DMFS) in Non-Small Cell Lung Cancer (NSCLC), isolated locoregional recurrences have increased. However, management of locoregional recurrences can be challenging. We report our institutional experience with definitive intent re-irradiation using Intensity Modulated Proton Therapy (IMPT).

**Method:**

Retrospective cohort study of recurrent or second primary NSCLC or LS-SCLC treated with IMPT. Kaplan-Meier method and log-rank test were used for time-to-event analyses.

**Results:**

22 patients were treated from 2019 to 2021. After first course of radiation (median 60 Gy, range 45-70 Gy), 45% received adjuvant immunotherapy. IMPT re-irradiation began a median of 28.2 months (8.8-172.9 months) after initial radiotherapy. The median IMPT dose was 60 GyE (44-60 GyE). 36% received concurrent chemotherapy with IMPT and 18% received immunotherapy after IMPT. The median patient’s IMPT lung mean dose was 5.3 GyE (0.9-13.9 GyE) and 5 patients had cumulative esophagus max dose >100 GyE with 1-year overall survival (OS) 68%, 1-year local control 80%, 1-year progression free survival 45%, and 1-year DMFS 60%. Higher IMPT (HR 1.4; 95% CI 1.1-1.7, p=0.01) and initial radiotherapy mean lung doses (HR 1.3; 95% CI 1.0-1.6, p=0.04) were associated with worse OS. Two patients developed Grade 3 pneumonitis or dermatitis, one patient developed Grade 2 pneumonitis, and seven patients developed Grade 1 toxicity. There were no Grade 4 or 5 toxicities.

**Discussion:**

Definitive IMPT re-irradiation for lung cancer can prolong disease control with limited toxicity, particularly in the immunotherapy era.

## Introduction

As improved systemic therapy leads to prolonged survival in lung cancer, the importance of locoregional disease control increases. The PACIFIC trial showed an unprecedented 5-year overall survival (OS) of 42.9% for unresectable non-small cell lung cancer (NSCLC) treated with definitive chemoradiation and adjuvant durvalumab, a PD-L1 inhibitor (1–3). Nevertheless, locoregional recurrences or second primary tumors after radiotherapy remain a therapeutic challenge (4, 5). Prior to immunotherapy use in locally advanced NSCLC, disease progression was common (6), but isolated locoregional recurrence rates after chemoradiotherapy or surgery were reported as low as 14-18% (1, 2, 7). However, on the PACIFIC trial, isolated thoracic progression occurred in 36.6% of patients (8). In the absence of metastatic disease, systemic therapy alone has limited chance of cure without local therapy (9). However, salvage re-irradiation or surgery can be toxic with questionable efficacy given inability to eradicate initial disease (7, 10).

Historically, re-irradiation was dangerous, with up to 14% Grade 5 toxicity (10, 11). This limited providers to lower, safer doses with limited efficacy beyond symptom palliation (4). At high doses, re-irradiation can risk fatal hemorrhage, myelopathy, brachial plexopathy, and even fatal pneumonitis (12–14). Yet, modern advances in radiation delivery have allowed better sparing of normal tissue (15, 16). Proton therapy, in particular, offers a dosimetric advantage in sparing normal tissue by minimizing exit dose that can enable delivery of curative intent re-irradiation doses (17–19). While most prior reports of proton therapy in the thorax employed passive scatter technique, intensity-modulated proton therapy (IMPT) is more conformal and enables lower doses to the normal lung, heart, and esophagus with subsequent lower rates of toxicity (20). This is especially important given the compound toxicity when immunotherapy is added to chemoradiation. Though now standard of care, the combination has been demonstrated to have higher rates of pneumonitis and other adverse events (2). In this work, we evaluated the safety and efficacy of definitive intent re-irradiation using IMPT in the era of immunotherapy.

## Materials and methods

This was a single-institution retrospective cohort study. De-identified demographic, treatment, and outcomes data were gathered, and informed consent was waived in accordance with institutional review board policies. Second primary tumors were defined by multi-disciplinary consensus for lesions with distinct histology (e.g. adenocarcinoma and squamous cell carcinoma) or based on timing and location of second tumor relative to initial disease. We included patients with locally advanced or unresectable, recurrent or second primary NSCLC or Limited Stage SCLC were treated with definitive-intent IMPT re-irradiation (doses in Gy equivalent [GyE]) using Varian ProBeam to overlapping areas. Patients were simulated for IMPT with motion management using either 4D-CT or average of 3 scans using SDX gated breathold technique. Treatment plans were generated using gross tumor with 5 mm Clinical Target Volume (CTV) margin cropped out of heart and esophagus. Plans were robustly optimized with setup error of 5 mm and range uncertainty of 5%. We evaluated covariates including age, Eastern Cooperative Oncology Group (ECOG) performance status, recurrent T-stage and N-stage per American Joint Committee on Cancer 8^th^ edition, use of concurrent chemotherapy with re-irradiation, immunotherapy use, time between radiation courses, and dosimetric variables from initial and second radiation courses (heart mean dose, lung V20 Gy or V20 GyE, lung mean dose, esophagus mean dose, esophageal max dose, aorta max dose, pulmonary artery max dose, proximal bronchial tree max dose, and brachial plexus max dose). Additionally we evaluated cumulative V15 Gy to the left anterior descending artery. All patients were treated in initial and re-irradiation courses with once daily radiotherapy. Outcomes assessed included overall survival (OS), local control (LC), progression-free survival (PFS), distant metastasis-free survival (DMFS), and any grade toxicity per Common Terminology Criteria for Adverse Events (CTCAE) version 5.0. Time-to-event endpoints were measured from date of re-irradiation completion. Patients were censored at last follow-up or, in the case of LC, at time of distant progression or death.

There were multiple patient-reported prospectively gathered quality of life instruments gathered on an institutional review board approved registry study with informed patient consent. The EQ-5D-5L is a validated 5-dimension assessment of quality of life with 5-levels (21). We report the results from the index value that summarizes all dimensions and the visual analogue scale (VAS) of overall self-reported health. The MD Anderson Dysphagia Index (MDADI) was used to assess dysphagia during and after re-irradiation. The MDADI scores ranges from 20-100 on subscales including functional, physical, emotional, and global domains (22). We report the composite score (functional, physical, and emotional scales) and the global subscale score. The MD Anderson Symptom Inventory (MDASI) with lung cancer specific symptoms was used (23). We report the core symptom score that is a composite of 13 common cancer and treatment related symptoms, the interference with daily function, and the symptom burden including lung cancer specific symptoms. In general, for all of these scales, higher scores represent better quality of life or lower symptom burden.

Data analyses were done in SAS^®^ 9.4 (Cary, NC) with SAS^®^ macros (24). The significance level was set *a priori* at p<0.05. Descriptive statistics for each variable were reported along with quality-of-life metrics. The Kaplan-Meier method was used to plot and estimate median and 1-year rates for OS, LC, PFS, and DMFS. The association with OS, LC, PFS, and DMFS were modeled by the log-rank test.

## Results

### Patient and tumor characteristic

We identified 22 patients treated with re-RT from April 2019 to May 2021 with recurrent (77.3%) or second primary (22.7%) lung cancers. Second primary lung cancers were defined as biopsied lesions with distinct histopathology from initial primary tumor based on pathologist’s evaluation. The median age was 71 years old (range 54-85 years old), and half of all patients had squamous cell carcinoma histology (n=11). Demographic details are outlined in **Table 1**. Of 9 patients tested for targetable mutations, only 1 had a positive result (EGFR Exon 20 mutation). That patient did not receive targeted therapy by last follow-up.

**Table 1:** Baseline patient demographics.

Variable	Level	Number	Percentage
Age	< 65 years old	4	18.2%
	≥ 65 years old	18	81.8%
Sex	Male	15	68.2%
	Female	7	31.8%
ECOG	0	4	18.2%
	1	15	68.2%
	2	3	13.6%
Histology	Squamous Cell Carcinoma	11	50.0%
	Adenocarcinoma	8	36.4%
	Small Cell Lung Cancer	2	9.1%
	Undifferentiated Carcinoma	1	4.5%
Relapse T-Stage	0	6	27.3%
	1	3	13.6%
	2	2	9.1%
	3	6	27.3%
	4	5	22.7%
Relapse N-Stage	0	9	40.9%
	1	3	13.6%
	2	6	27.3%
	3	4	18.2%
Relapse Group Stage	1	2	9.1%
	2	8	36.4%
	3A	4	18.2%
	3B	7	31.8%
	3C	1	4.5%
PD-L1 Status	<1%	4	44.4%
	≥1%	5	55.6%
	Missing	13	–
Actionable Mutation	Yes	1	88.9%
	No	8	11.1%
	Missing	13	–

### Initial and re-irradiation treatment details

Initial, curative intent photon treatment was stereotactic ablative radiotherapy (n=2) or chemoradiation (n=20). The most common initial prescription was 60-66 Gy conventionally fractionated at 2 Gy per fraction (63.6%, n=14). The dosimetric details of the initial radiation courses are summarized in **Table 2**. After initial radiotherapy, 45.5% (n=10) of patients received Durvalumab.

**Table 2:** Radiation treatment characteristics.

Variable	Initial Course Median (Range)	Initial Course BED for α/β = 3 (Range)	IMPT Median (Range)	IMPT BED for α/β = 3 (Range)	Cumulative Median (Range)
Prescription Dose	60 Gy (45-70 Gy)		60 GyE (44-60 GyE)		120 GyE (89-130 GyE)
Lung V20 Gy (or GyE)	19% (1%-3%)		8% (2%-26%)		22% (7%-54%)
Lung V5 Gy (or GyE)	51% (4%-96%)		17% (3%-46%)		55% (13%-99%)
Mean Lung dose	12 Gy (1-20 Gy)	14 Gy (1-26 Gy)	4 GyE (1-14 GyE)	5 GyE (1-17 GyE)	17 GyE (5-33 GyE)
Heart V10 Gy (or GyE)	17% (0%-89%)		3% (0%-51%)		22% (4%-89%)
Mean Heart dose	6 Gy (1-25 Gy)	6 Gy (1-33 Gy)	1 GyE (0-24 GyE)	1 GyE (0-31 GyE)	9 GyE (2-54 GyE)
Max Spinal Cord dose	29 Gy (5-46 Gy)	40 Gy (5-86 Gy)	10 GyE (0-35 GyE)	11 GyE (0-50 GyE)	41 GyE (5-58 GyE)
Mean Esophagus dose	18 Gy (2-50 Gy)	21 Gy (2-72 Gy)	4 GyE (0-24 GyE)	4 GyE (0-31 GyE)	31 GyE (2-62 GyE)
Max Aorta dose	59 Gy (17-72 Gy)	107 Gy (36-130 Gy)	47 GyE (0-63 GyE)	93 GyE (0-147 GyE)	92 GyE (24-133 GyE)
Max Pulmonary Artery dose	57 Gy (0-69 Gy)	102 Gy (0-122 Gy)	47 GyE (0-62 GyE)	90 GyE (0-120 GyE)	87 GyE (0-127 GyE)
Max Proximal Bronchial Tree dose	64 Gy (8-70 Gy)	110 Gy (9-124 Gy)	63 GyE (6-64 GyE)	105 GyE (0-155 GyE)	110 GyE (13-129 GyE)
Max Brachial Plexus dose	34 Gy (0-71 Gy)	49 Gy (0-127 Gy)	7 GyE (0-62 GyE)	8 GyE (0-147 GyE)	37 GyE (0-132 GyE)

BED, biologically equivalent dose; IMPT, intensity modulated proton therapy.

Curative intent IMPT was initiated a median of 28 months after initial photon radiotherapy (range 9 – 173 months). The majority of patients were treated free breathing (91%, n=20). Concurrent platinum-based chemotherapy was administered to 36% of patients, most commonly carboplatin and paclitaxel. Re-irradiation was most frequently prescribed to 60 GyE (68.2%) and the two most common fractionations were 30 fractions (50.0%) or 15 fractions (31.8%). The dosimetric details of IMPT re-irradiation are summarized in **Table 2**. Two patients stopped treatment after 22 and 29 fractions of planned 30-fraction regimens. One patient refused chemotherapy and was replanned for hypofractionation during the second half of their treatment. Five patients had cumulative esophagus max doses of >100 GyE (overall median 89 GyE, range 17-111 GyE). The median cumulative V15 GyE to the Left Anterior Descending coronary artery was 35% (range 0%-100%). A total of 5 patients (23%) had V15 GyE < 10%. After re-irradiation with IMPT, 18% of patients received pembrolizumab or durvalumab.

### Outcomes and associations

**Figure 1:**
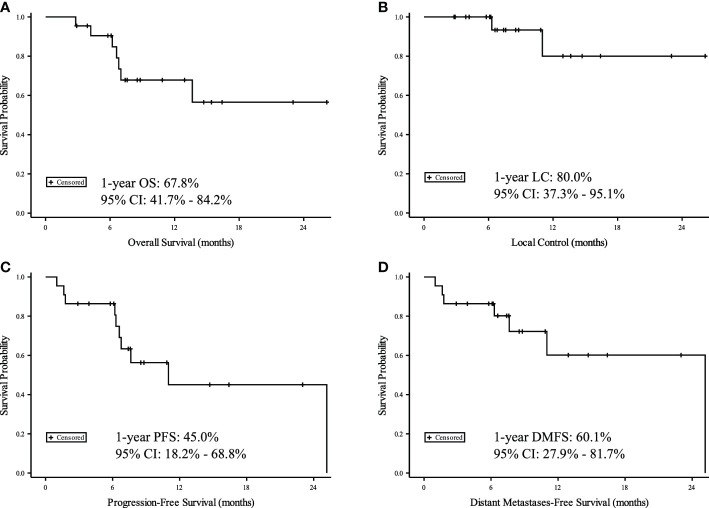
Kaplan-Meier curves for 22 patients with recurrent or second primary lung cancers. Time measured from date of IMPT for **(A)** Overall Survival (OS), **(B)** Local Control (LC), **(C)** Progression Free Survival (PFS), and **(D)** Distant Metastasis Free Survival (DMFS).

After median follow-up of 7 months (range 3-26 months) from completion of IMPT, 1-year OS was 68%; 1-year LC was 80%; 1-year PFS was 45%; and 1-year DMFS was 60% (**Figure 1**). There were no Grade 4 or 5 toxicities. Two patients (9%) had Grade 3 toxicity (bronchial necrosis and skin desquamation with fibrosis). One patient (5%) had Grade 2 pneumonitis. Seven patients (32%) had Grade 1 fatigue, cough, skin reactions, esophagitis, or pneumonitis. Toxicities summarized in **Supplemental Table 1**. On review of cumulative dose distribution for the patient with bronchial necrosis, the right mainstem bronchus D1cc was 126.3 GyE and D0.03cc was 130.1 GyE.

On univariate analysis, lower total IMPT completed dose (HR: 4.59, 95% CI: 0.99-21.29, p=0.03), higher initial RT lung mean dose (HR: 1.26, 95% CI: 1.01-1.56, p=0.04), and higher IMPT lung mean dose (HR 1.38, 95% CI: 1.09-1.74, p<0.01) were all associated with worse OS. In addition, there was trend toward worse OS with higher IMPT heart mean dose (HR 1.29, 95% CI: 0.98-1.68, p=0.07) and higher cumulative esophagus max dose (HR 1.06, 95% CI: 1.00-1.13, p=0.06). There were no significant associations with LC. Higher IMPT mean lung dose correlated with worse PFS (HR 1.25, 95% CI: 1.03-1.51, p=0.02) on univariate analysis. There was also a trend toward worse PFS with higher initial RT lung mean dose (HR 1.15, 95% CI: 0.98-1.33, p=0.08), IMPT heart mean dose (HR 1.10, 95% CI: 1.00-1.22, p=0.06), and higher cumulative esophagus max dose (HR 1.05, 95% CI: 1.00-1.09, p>0.05). There were no significant associations with DMFS, though higher IMPT heart mean dose trended towards worse DMFS (HR 1.10, 95% CI: 0.99-1.22, p=0.07).

Univariate associations with toxicity were assessed for patient, tumor, and treatment characteristics. There were no significant associations with age (p=0.55), performance status (p=1.0), recurrent T-stage (p=1.0) or N-stage (p=0.57), time between initial RT and IMPT (p=0.32), completion of at least 50 GyE IMPT (p=0.57), use of ≥20 fractions (p=0.38), or use of concurrent chemotherapy (p=1.0) or adjuvant immunotherapy (p=1.0). There were no significant associations with any dosimetric features and subsequent toxicity including IMPT lung V20 (p=0.40), IMPT lung mean dose (p=0.39), or cumulative esophagus max dose (p=0.63). Complete associations of patient, tumor, and treatment characteristics with OS, LC, PFS, and DMFS are summarized in **Supplemental Table 2**.

### Quality of life

At baseline, 19 patients completed EQ-5D-5L with median Index score 0.84 (range 0.23-1.0) and median VAS score 51 (range 22-100). In follow-up, 11 patients completed EQ-5D-5L and at the last follow-up, median change in index score was +0.04 (range -0.60 to +0.38), and the median change in VAS score was 0 (range -48 to +58).

At baseline, 20 patients completed the MDADI with median composite score 81 (range 45-100) and median global score 80 (range 20-100). In follow-up, 18 patients completed the MDADI. At the last follow-up, the median change in composite score was -0.5 (range -37 to +23), and the median change in global score was 0 (range -20 to +20).

At baseline, 20 patients completed the MDASI with median core score 2.5 (range 0-7.5), median interference score 2.5 (range 0-8.5), and median symptom score 2.2 (range 0.8-2.9). In follow-up, 11 patients completed the MDASI. At the last follow-up, the median change in core score was 0 (range -1.8 to +2.9), the median change in interference score was -1.1 (range -4.7 to +2.8), and the median change in symptom score was +0.2 (range -2.1 to +2.8).

**Figure 2:**
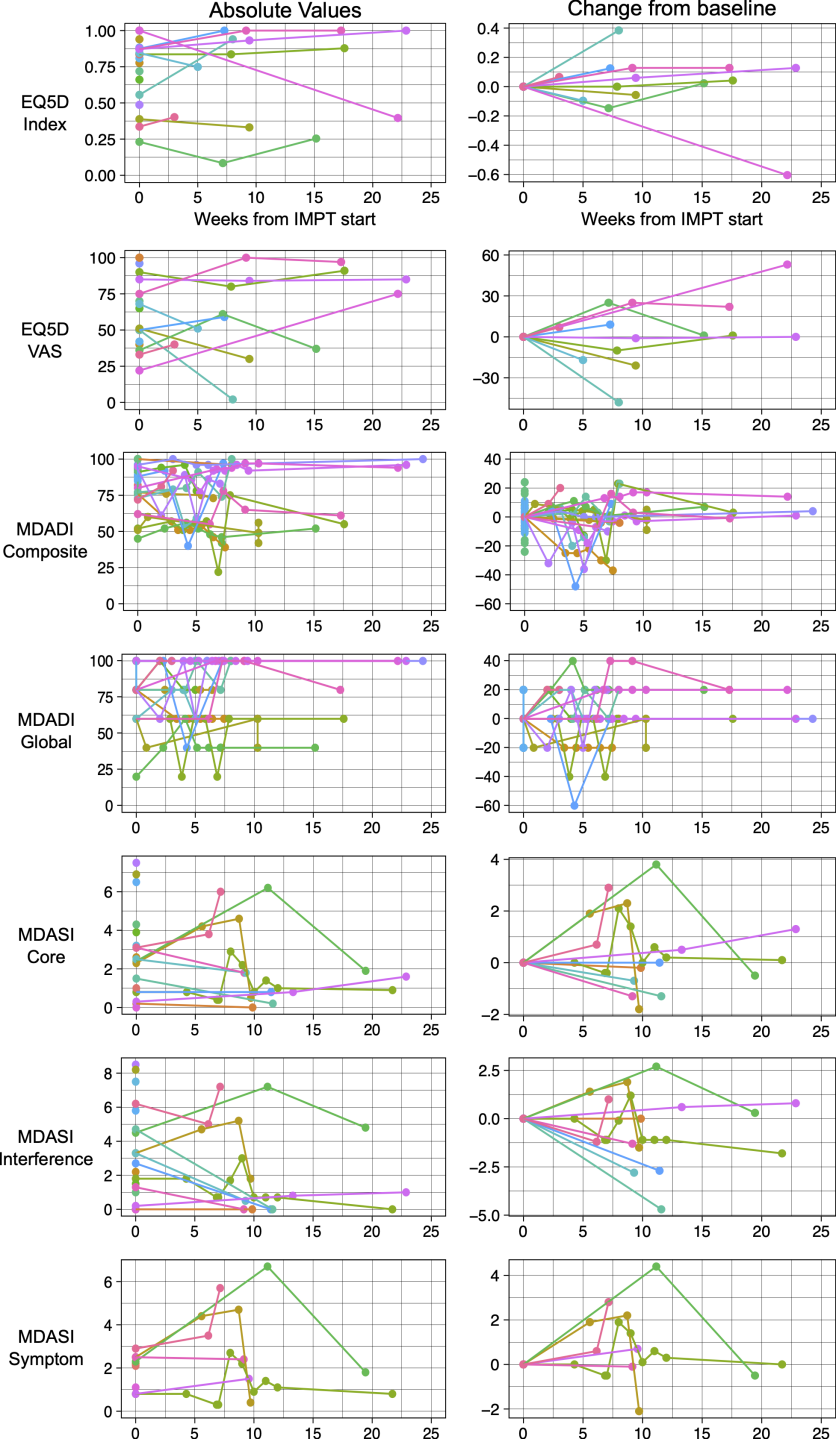
Patient reported quality of life instruments including EQ-5D-5L, MD Anderson Dysphagia Index (MDADI) and MD Anderson Symptom Inventory (MDASI). Patient scores over time depicted on the left and change from baseline depicted on the right.

A summary of the first 25 weeks of quality-of-life scores and change from baseline is depicted in **Figure 2**.

## Discussion

Thoracic re-irradiation has historically been limited due to the risk of toxicity. IMPT has emerged as a new radiation modality that better spares normal tissue and thus may alter the therapeutic ratio. In this study, we showed that for 22 patients with locoregionally recurrent or second primary NSCLC or SCLC that definitive intent IMPT was safe and effective with 1-year LC and OS rates of 80% and 68% respectively. Despite 5 patients receiving cumulative esophageal max dose >100 GyE and some patients receiving mean heart dose >20 GyE, we observed no Grade 4 or 5 toxicities and minimal changes on a variety of quality-of-life metrics. However, these were selected patients with good performance status and a prolonged interval between radiation treatments that received highly conformal IMPT. Isolated thoracic recurrence has become the dominant pattern of disease recurrences in the post-PACIFIC era (8), and our results suggest that thoracic re-irradiation can offer a safe and effective treatment with potential opportunity for cure.

Salvage re-irradiation decisions are predominantly guided by heterogeneous retrospective data. Sources typically include 20-50 patients but vary from case reports (25) to hundreds of patients (26). These cohorts can focus on patients with early stage disease at initial presentation (26) or recurrence (27) to cohorts that are exclusively locally advanced (28, 29). Treatment in these reports varies from salvage SBRT (26–28, 30–32) to fractionated radiation with or without chemotherapy (14, 29, 33–36). As such, outcomes vary from median OS of 9.3 months (29) to 37 months (26). The efficacy of definitive intent re-irradiation has been relatively high with prior reports showing local recurrence rates below 20%, though most of those reports include favorable disease with patients eligible for SBRT. However, there are very few reports of IMPT in the re-irradiation setting (36), with most of the prior proton re-irradiation literature employing passive scatter techniques and even fewer that report quality of life. With IMPT we observed 80% LC at 1-year with the majority of patients who progressed doing so distantly, median OS not yet reached, and minimal changes in quality-of-life metrics (with multiple patients reporting improvements). With appropriate patient selection, re-irradiation can provide a chance of cure. Patients with long interval between initial radiation and relapse, initial treatment that met all constraints for thoracic organs at risk, and who did not experience Grade 2+ pneumonitis with initial treatment are good candidates for re-irradiation.

Upfront treatment of locally advanced NSCLC can be toxic, with 76% of patients on RTOG 0617 experiencing Grade 3+ adverse events (37). Re-irradiation can be even more dangerous. The rate of Grade 5 toxicity with re-irradiation has been reported to be as high as 10% in multiple studies using either protons (38) or photons (35, 39). Predictors of Grade 3+ pneumonitis have included worse performance status and cumulative lung V20 > 30% (27, 40). While there are not clear dosimetric correlates to predict cardiac toxicity in the re-irradiation setting (5, 40), V15 >10% to the left anterior descending artery has been associated with increased major cardiovascular events and all-cause mortality (41). The majority of our patients (77%) had cumulative V15 >10%. However, high point doses to central mediastinal structures can be the most dangerous part of upfront SBRT or re-irradiation regardless of fractionation. Risk of aortic rupture has been as high as 25% when cumulative doses exceed 120 Gy (14). Fortunately we observed no aortic toxicity despite 4 patients exceeding cumulative nominal dose of 120 GyE. After two Grade 5 bleeds on a Phase II trial of chemoradiation followed by SBRT for local recurrence, Feddock et al. recommended constraining pulmonary artery and bronchial wall to 185 Gy and 175 Gy BED_3_ or EQD2 of 111 Gy and 105 Gy respectively (42). Notably, our patient with Grade 3 bronchial necrosis had a cumulative D0.03cc of 130.1 GyE and their IMPT re-irradiation was prescribed to 60 GyE in 15 fractions, the highest BED of any patient in our cohort. Brachial plexopathy rarely happens with Dmax below 80 Gy, but can occur in up to 47% with Dmax ≥ 95 Gy (43). With four patients having cumulative nominal Dmax >95 Gy to the brachial plexus, we observed no plexopathy in follow-up. Similarly, spinal constraints of <67.5 Gy in 2 Gy per fraction equivalent cumulative dose are generally well-tolerated assuming >6 months between radiation (44, 45). In general, our results show that patients with higher doses to thoracic organs at risk from initial treatment had worse outcomes, highlighting the challenges of patient selection for who might benefit most from definitive intent re-irradiation. Furthermore, patients with higher lung mean dose with IMPT re-irradiation also had worse outcomes, underscoring potential importance of highly conformal radiation.

Our study is subject to limitations, including those inherent to a single institution retrospective study. Notably, the generalizability of our findings is limited primarily by cohort size and heterogeneity of histologies, time between radiation, and systemic therapies (e.g. concurrent chemotherapy or immunotherapy use). However, the initial and re-irradiation prescriptions were fairly standardized in our cohort. Confounding between bulk of initial and recurrent disease with dosimetric variables such as lung mean dose could account for worse outcomes in patients with higher lung mean doses, particularly as there were not enough patients to run multivariable analyses. Additionally, given the small number of Grade 3 toxicity or local progression events, Type II errors in our analyses are possible since underpowered to observe correlations with low-incidence events. Furthermore, due to infeasibility, patients deemed to be too high risk for re-irradiation by referring or treating providers are not included as a comparator arm to assess dosimetric features or outcomes. The quality-of-life data is incomplete and non-standardized in timings of assessments, limiting statistical inferences from this cohort.

Future directions include prospective assessments of re-irradiation as well as pooling data to better characterize constraints to prevent high grade toxicities. While cooperative group studies are exploring innovations to optimize upfront treatment (46–49), there are at least two ongoing Phase II trials examining SBRT or fractionated chemoradiation for thoracic recurrences. Sun Yat-sen University is assessing SBRT for recurrences that are both peripheral (50-60 Gy in 10 fractions) and central (30-40 Gy in 6-10 fractions followed by adaptive re-planned 24-35 Gy boost in 4-7 fractions after 4 week break) (50). The University of Pennsylvania is assessing IMPT chemoradiation followed by pembrolizumab for locoregionally recurrent NSCLC (51). Clinical and pre-clinical data continues to be published on optimizing immunotherapy and combination of radiation and immunotherapy (52–56). Outside of prospective trials, pooled retrospective data could allow for stronger inferences into dosimetric correlates with toxicity. As systemic therapy continues to improve control of extrathoracic lesions, the importance of safe local therapies to provide durable regional control will increase.

Patients who receive definitive IMPT re-irradiation for NSCLC and SCLC can experience prolonged disease control with limited toxicity or detriment to quality of life, particularly in the modern era with regular use of PD-L1 immune checkpoint inhibitors. However, careful patient selection and planning are needed for optimal outcomes, particularly preservation of sufficient functional lung.

## Data availability statement

The raw data supporting the conclusions of this article will be made available by the authors, without undue reservation.

## Ethics statement

The studies involving human participants were reviewed and approved by Emory University Institutional Review Board. Written informed consent for participation was not required for this study in accordance with the national legislation and the institutional requirements.

## Author contributions

JJ-N – Conceptualization, Methodology, Formal Analysis, Investigation, Data Curation, Original Drafting, Revising, and Editing. YC – Methodology, Formal Analysis, Visualization, Revising, and Editing. NM – Conceptualization, Methodology, Revising, and Editing. JMS – Methodology, Formal Analysis, Investigation, Visualization, Revising and Editing. ST – Revising and Editing. HC – Data Curation, Revising and Editing. WS – Revising and Editing. AK – Revising and Editing. MM – Resources, Revising and Editing. JWS – Revising and Editing. JB – Resources, Supervision, Revising and Editing. KH – Conceptualization, Methodology, Investigation, Original Drafting, Revising, and Editing. All authors contributed to the article and approved the submitted version.
