# Supplementary material for: Definitive intensity modulated proton re-irradiation for lung cancer in the immunotherapy era
Source: Front Oncol. 2023 Jan 17;12:1074675. doi: 10.3389/fonc.2022.1074675 (PMC9888533; doi:10.3389/fonc.2022.1074675)
Supplement: Supplementary file 1 [file DataSheet_1.docx]

| **Supplemental Table 1** | | |  |
| --- | --- | --- | --- |
| Toxicity | Grade 1 (%) | Grade 2 (%) | Grade 3 (%) |
| Bronchial Necrosis | - | - | 1 (5%) |
| Cough | 3 (14%) | - | - |
| Dermatitis | - | - | 1 (5%) |
| Esophagitis | 2 (9%) | - | - |
| Fatigue | 1 (5%) | - | - |
| Neuropathy | 1 (5%) | - | - |
| Pneumonitis | 1 (5%) | 1 (5%) | - |

| **Supplemental Table 2** | | | | | | | | | | |
| --- | --- | --- | --- | --- | --- | --- | --- | --- | --- | --- |
| Variable | Level | n | OS | | LC | | PFS | | DMFS | |
|  |  |  | HR (95% CI) | p | HR (95% CI) | p | HR (95% CI) | p | HR (95% CI) | p |
| Age | continuous | 22 | 0.99 (0.89-1.11) | 0.92 | 1.29 (0.81-2.06) | 0.29 | 0.99 (0.90-1.08) | 0.76 | 1.00 (0.89-1.13) | 0.98 |
| Age | <70 | 10 | 2.88 (0.62-13.28) | 0.18 | - | 0.34 | 2.91 (0.68-12.40) | 0.15 | 1.84 (0.35-9.55) | 0.47 |
|  | >70 | 12 | - |  | - |  | - |  | - |  |
| Time between radiation | continuous | 22 | 1.00 (0.98-1.02) | 0.85 | 1.00 (0.97-1.03) | 0.99 | 1.00 (0.98-1.01) | 0.85 | 1.00 (0.99-1.02) | 0.70 |
| ECOG | 0 | 4 | - | 0.23 | 4.03 (0.23-69.78) | 0.30 | 0.49 (0.06-4.04) | 0.50 | 0.76 (0.09-6.61) | 0.80 |
|  | 1-2 | 18 | - |  | - |  | - |  | - |  |
| Relapse T-stage | 0-2 | 11 | 0.39 (0.08-2.05) | 0.25 | 1.08 (0.07-17.54) | 0.96 | 0.55 (0.14-2.19) | 0.39 | 0.49 (0.09-2.69) | 0.40 |
|  | 3-4 | 11 | - |  | - |  | - |  | - |  |
| Relapse N-stage | 0-1 | 18 | - | 0.19 | - | 0.42 | 1.98 (0.25-15.90) | 0.51 | - | 0.19 |
|  | 2-3 | 4 | - |  | - |  | - |  | - |  |
| Concurrent chemotherapy | Yes | 8 | 1.21 (0.27-5.46) | 0.80 | 1.41 (0.09-22.64) | 0.81 | 1.39 (0.37-5.23) | 0.63 | 1.60 (0.32-7.98) | 0.56 |
|  | No | 14 | - |  | - |  | - |  | - |  |
| Immunotherapy after IMPT | Yes | 4 | 0.51 (0.06-4.49) | 0.54 | - | 0.32 | 0.38 (0.05-3.08) | 0.35 | 0.64 (0.07-5.57) | 0.68 |
|  | No | 18 | - |  | - |  | - |  | - |  |
| IMPT completed dose | <50 GyE | 4 | 4.59 (0.99-21.29) | **0.03** | - | 0.57 | 2.84 (0.70-11.50) | 0.13 | 1.16 (0.13-9.97) | 0.89 |
|  | > 50 GyE | 18 | - |  | - |  | - |  | - |  |
| Initial RT lung mean dose | continuous | 22 | 1.26 (1.01-1.56) | 0.04 | 1.03 (0.77-1.37) | 0.85 | 1.15 (0.98-1.33) | 0.08 | 1.12 (0.93-1.34) | 0.22 |
| Initial RT lung mean dose | <15 Gy | 14 | 0.34 (0.08-1.52) | 0.157 | 0.38 (0.02-6.10) | 0.50 | 0.55 (0.15-2.08) | 0.38 | 0.45 (0.09-2.30) | 0.34 |
|  | >15 Gy | 8 | - |  | - |  | - |  | - |  |
| Initial RT lung V20 Gy | <20% | 13 | 0.43 (0.10-1.93) | 0.272 | 0.61 (0.03-9.93) | 0.73 | 0.44 (0.12-1.65) | 0.22 | 0.65 (0.13-3.26) | 0.61 |
|  | >20% | 9 | - |  | - |  | - |  | - |  |
| Initial RT heart mean dose | continuous | 22 | 1.07 (0.96-1.20) | 0.22 | 1.13 (0.90-1.42) | 0.31 | 1.07 (0.98-1.18) | 0.16 | 1.06 (0.94-1.18) | 0.35 |
| Initial RT heart mean dose | <10 Gy | 14 | 0.59 (0.13-2.72) | 0.503 | - | 0.18 | 0.37 (0.09-1.51) | 0.17 | 0.41 (0.07-2.28) | 0.31 |
|  | >10 Gy | 8 | - |  | - |  | - |  | - |  |
| IMPT lung mean dose | continuous | 22 | 1.38 (1.09-1.74) | **<0.01** | 0.71 (0.33-1.52) | 0.38 | 1.25 (1.03-1.51) | **0.02** | 1.12 (0.90-1.40) | 0.32 |
| IMPT lung mean dose | <5 GyE | 12 | 0.27 (0.05-1.38) | 0.12 | - | 0.16 | 0.33 (0.08-1.32) | 0.12 | 0.78 (0.16-3.91) | 0.77 |
|  | >5 GyE | 10 | - |  | - |  | - |  | - |  |
| IMPT lung V20 GyE | <15% | 16 | 0.12 (0.03-0.57) | **0.007** | - | 0.57 | 0.22 (0.05-0.93) | **0.04** | 0.40 (0.06-2.57) | 0.34 |
|  | >15% | 6 | - |  | - |  | - |  | - |  |
| IMPT heart mean dose | continuous | 22 | 1.29 (0.98-1.68) | 0.07 | 1.28 (0.60-2.72) | 0.53 | 1.10 (1.00-1.22) | 0.06 | 1.10 (0.99-1.22) | 0.07 |
| Cumulative esophagus max dose | continuous | 22 | 1.06 (1.00-1.13) | 0.06 | 0.98 (0.93-1.04) | 0.54 | 1.05 (1.00-1.09) | >0.05 | 1.02 (0.98-1.06) | 0.37 |
| Univariate associations between patient, tumor, and treatment covariates with Overall Survival (OS), Local Control (LC), Progression Free Survival (PFS), and Distant Metastasis Free Survival (DMFS). Hazard Ratios (HR) and 95% Confidence intervals (95% CI) reported with HR p-values for continuous variables and log-rank p-values for categorical variables (p). HR omitted for too few events in one arm. RT = radiotherapy. IMPT = Intensity Modulated Proton Therapy. ECOG = Eastern Cooperative Oncology Group performance status. | | | | | | | | | | |
